# Crohn’s and Colitis Canada’s 2021 Impact of COVID-19 and Inflammatory Bowel Disease in Canada: COVID-19 Vaccines—Biology, Current Evidence and Recommendations

**DOI:** 10.1093/jcag/gwab033

**Published:** 2021-11-05

**Authors:** Sanjay K Murthy, M Ellen Kuenzig, Joseph W Windsor, Jean-Eric Ghia, Anne M Griffiths, Remo Panaccione, Cynthia H Seow, Eric I Benchimol, Charles N Bernstein, Alain Bitton, James Guoxian Huang, Jennifer L Jones, Kate Lee, Gilaad G Kaplan, Mariam S Mukhtar, Parul Tandon, Laura E Targownik, Deanna L Gibson

**Affiliations:** 1 The Ottawa Hospital IBD Centre, Department of Medicine, University of Ottawa, Ottawa, Ontario, Canada; 2 SickKids Inflammatory Bowel Disease Centre, Division of Gastroenterology, Hepatology and Nutrition, The Hospital for Sick Children, Toronto, Ontario, Canada; 3 Child Health Evaluative Sciences, SickKids Research Institute, Toronto, Ontario, Canada; 4 Department of Medicine, University of Calgary, Calgary, Alberta, Canada; 5 Department of Immunology & Internal Medicine section of Gastroenterology, Max Rady College of Medicine, Rady Faculty of Health Sciences, University of Manitoba and University of Manitoba Inflammatory Bowel Disease Clinical and Research Centre, Winnipeg, Manitoba, Canada; 6 ICES, Toronto, Ontario, Canada; 7 Department of Paediatrics and Institute of Health Policy, Management and Evaluation, University of Toronto, Toronto, Ontario, Canada; 8 Division of Gastroenterology and Hepatology, Department of Medicine, University of Calgary, Calgary, Alberta, Canada; 9 Department of Community Health Sciences, University of Calgary, Calgary, Alberta, Canada; 10 Department of Internal Medicine, Max Rady College of Medicine, Rady Faculty of Health Sciences, University of Manitoba, Winnipeg, Manitoba, Canada; 11 University of Manitoba IBD Clinical and Research Centre, Winnipeg, Manitoba, Canada; 12 Department of Medicine, McGill University Health Centre, McGill University, Quebec, Canada; 13 Department of Medicine, Dalhousie University, Halifax, Nova Scotia, Canada; 14 Crohn’s and Colitis Canada, Toronto, Ontario, Canada; 15 Department of Internal Medicine, King Abdulaziz University Hospital, Jeddah, Saudi Arabia; 16 Division of Gastroenterology and Hepatology, Mount Sinai Hospital, University of Toronto, Toronto, Ontario, Canada; 17 Department of Biology, Faculty of Science; Department of Medicine, Faculty of Medicine, The University of British Columbia, Okanagan campus, Kelowna, British Columbia, Canada

**Keywords:** COVID-19, Crohn’s disease, Inflammatory bowel disease, SARS-CoV-2, Ulcerative colitis, Vaccination, Vaccine

## Abstract

The COVID-19 pandemic has ushered a globally focused vaccine development program that produced multiple successful vaccines within a year. Four SARS-CoV-2 vaccines have been approved for use in Canada, using two different technologies, all of which have shown excellent efficacy in reducing the rate of symptomatic COVID-19 infection and 100% efficacy in preventing death from COVID-19. People with inflammatory bowel disease (IBD), like many others with immune-mediated chronic diseases, were excluded from the pivotal trials of these vaccines, leading to early hesitancy by regulatory bodies to endorse administering the vaccines to these groups. However, recent data has shown that the adverse event rate to SARS-CoV-2 vaccine among people with IBD is similar to the general population. Early data has further shown that people with IBD are capable of mounting a robust immune response to SARS-CoV-2 vaccines, particularly following a second dose, whereas the response to the first dose is blunted in those receiving anti-TNF therapy or conventional immunosuppressants (azathioprine, 6-mercaptopurine, methotrexate). Based on these data and evidence from previous vaccine programs among people with IBD, multiple national and international expert panels have recommended that individuals with IBD receive complete vaccination against SARS-CoV-2 as soon as possible.

**Key Messages**

- Although people with IBD were excluded from the SARS-CoV-2 vaccine trials, real-world evidence suggests that these vaccines are safe and elicit robust immune responses and protect against SARS-CoV-2 infection following two doses of mRNA vaccine.
- The effectiveness of the first dose of vaccine (for a two-dose vaccine) is limited (reduced immunogenicity) for people with IBD receiving anti-TNF therapy or conventional immunosuppressants (e.g., prednisone, azathioprine, 6-mercaptopurine, methotrexate).
- National and international expert panels recommend that all people with IBD receive a complete series SARS-CoV-2 vaccine at the earliest opportunity, irrespective of vaccine type, disease status, or treatment.

## Introduction

In response to the COVID-19 pandemic, international pharmaceutical companies have rapidly developed, tested and produced highly effective vaccines against the Severe Acute Respiratory Syndrome CoronaVirus 2 (SARS-CoV-2) virus. More than 200 COVID-19 vaccine candidates are under development or in clinical trials, using both traditional (inactivated or live attenuated vaccines) and newer (recombinant protein vaccines, vectored vaccines and RNA and DNA vaccines) technologies (1,2). Four vaccines having now been approved for use in Canada (COVID-19 Vaccines: Authorized vaccines: Canada.ca).

Many individuals with immune-mediated chronic diseases, including all persons with inflammatory bowel disease (IBD), were excluded from the clinical trials that ultimately led to the approval of these vaccines. Thus, there was initially some uncertainty regarding efficacy and safety of these vaccines in people with IBD. This article reviews the immunology, mechanisms and evidence for currently available (as of writing: May 25, 2021) COVID-19 vaccines and discusses the potential risks and benefits of people with IBD being vaccinated.

## IMMUNITY AND COVID-19: NATURAL AND VACCINE IMMUNITY

Natural COVID-19 infection often results in exaggerated and dysfunctional innate and adaptive immune responses (3,4). Inflammasome activation and increases in inflammatory monocytes and neutrophils lead to an overexuberant cytokine storm of IL-1β, IL-6 and TNF-α, exacerbating severe vascular and respiratory insults (5). Indeed, late-stage pathology in COVID-19 is driven primarily by host immune responses to SARS-CoV-2 (4). Furthermore, evidence reveals T follicular helper cell differentiation may be faulty in individuals who develop lethal COVID-19 infection, given the lack of mature germinal centers and resultant impairment in the somatic hypermutation process required for class switching to IgG in these individuals (6). Conversely, some studies have found that convalescent patients have circulating T follicular helper memory cells, which are positively correlated with spike protein-specific neutralizing IgG, IgM and IgA antibodies (7). While neutralizing antibodies will afford protection against SARS-CoV-2, a misguided humoral response contributes to some of the immunopathology of COVID-19, including autoantibody production (8). These autoantibodies not only impair immune function and exacerbate COVID-19 severity but also have long-lasting potential for systemic autoimmune diseases post-infection. Improved understanding of the natural immune response will be instrumental in developing an effective vaccine and other therapeutic strategies.

Controlling COVID-19 requires effective and safe vaccines that provide disease-attenuating immunity, including robust T-cell and B-cell memory responses with the development of germinal centers and high-affinity neutralizing antibodies against COVID-19 and any variants. As variants of concern circulate in the population, several types of vaccines may become important in achieving herd immunity (when 70–95% of the population is immune) (9). Vaccine-induced protective immunity is safer and likely to be more effective than natural immunity. There is no evidence to suggest that T follicular helper cells or memory B cells responses would be blunted relative to natural infection or that vaccination would produce the plethora of maladaptive immune and autoimmune reactions seen with natural COVID-19 infection (e.g., cytokine storm or multisystem inflammatory syndrome in children). While some early research suggested immunity might be short-lived to SARS-CoV-2 infection, recent studies have shown that humoral response is long-lived and antigen-specific, including to the spike protein (10), providing good rational for using the spike protein as a vaccine candidate—as is the case with all currently approved SARS-CoV-2 vaccines in Canada.

## CURRENTLY APPROVED SARS-COV-2 VACCINES: MECHANISMS, EFFICACY AND SAFETY

Four SARS-CoV-2 vaccines have received Health Canada authorization for use in persons aged 18 years or older; one of these (Pfizer-BioNTech) has recently received approval for use in persons aged 12 and older (COVID-19 Vaccines: Authorized vaccines - Canada.ca): 1) Pfizer-BioNTech BNT162b2 mRNA vaccine (Pfizer, Inc., NY; BioNTech SE, Mainz, Germany); 2) NIH-Moderna mRNA-1273 vaccine (Moderna, Inc., Cambridge); 3) AstraZeneca ChAdOx1 nCoV-19 adenovirus vector vaccine (AstraZeneca plc, Cambridge, England) and 4) Janssen Ad26.COV2.S adenovirus vector vaccine (Janssen Pharmaceuticals, Beerse, Belgium). Large clinical trials have demonstrated these vaccines to be highly efficacious and safe (11–14). Mild side effects, such as injection site reactions (muscle pain, redness and swelling), fatigue, malaise, headaches, joint pains, low-grade fevers and chills were common in all trials. Serious adverse events were rare, and no deaths attributable to the vaccine were encountered in any trials. Importantly, the approved vaccines do not demonstrate the potential for virus activation or integration into the human genome.

## MESSENGER RNA (mRNA) VACCINES

The Pfizer-BioNtech vaccine (approved by Health Canada December 9, 2020) and the NIH-Moderna vaccine (approved by Health Canada December 23, 2020) use a lipid nanoparticle delivery system to transport modified SARS-CoV-2 genetic material (messenger ribonucleic acid [mRNA]) encoding the virus’ spike protein to host cells (11,12). The viral mRNA enters host cells and uses its translational machinery to produce copies of the spike protein, which then embed into the host’s cell membranes, prompting an adaptive immune response resulting in memory T and B lymphocytes producing neutralizing antibodies to the spike protein. These lymphocytes recognize and fight future SARS-CoV-2 infection faster and more effectively than their precursors. Both vaccines are administered as two intramuscular injections 21 (Pfizer) or 28 (Moderna) days apart. To vaccinate a maximum number of individuals amid a vaccine shortage, the National Advisory Council on Immunization (NACI) has recommended that the doses may be administered up to 16 weeks apart, based on suggestion of high-level protection against COVID-19 beyond 14 days following the first dose of either of these vaccines (11,12,15).

A large multinational RCT in 43,548 persons, 16 years of age or older who were healthy or had stable chronic disease (excluding immune-mediated inflammatory diseases) demonstrated 95.3% efficacy of the Pfizer-BioNtech vaccine in reducing symptomatic COVID-19 infections at least seven days after the second dose and 90% efficacy in reducing severe disease leading to hospitalization or death (one versus nine severe COVID-19 cases in vaccinated versus unvaccinated individuals) (11). A similarly designed RCT conducted in the US in 30,420 persons aged 18 years or older who were healthy or had stable chronic disease demonstrated 94.1% efficacy of the NIH-Moderna vaccine at least 14 days after the second dose and 100% efficacy in reducing severe disease leading to hospitalization or death (12). Recent data have shown these vaccines to be more than 90% effective out to six months without any serious safety concerns (16).

## ADENOVIRUS VECTOR VACCINES

The AstraZeneca vaccine (approved by Health Canada February 26, 2021) and the Janssen Ad26.COV2.S vaccine (approved by Health Canada on March 5, 2021) use a non-replicating adenovirus vector to deliver SARS-CoV-2 DNA encoding the spike protein to human cells, which uses the host machinery to produce viral spike protein (13). The AstraZeneca vaccine is administered as two intramuscular injections between four and 12 weeks apart, while the Janssen vaccine is administered as a single intramuscular injection.

A pooled interim analysis of 11,636 healthy adults randomized to either active vaccine or control across four RCTs held in the UK, Brazil and South Africa showed 66.7% efficacy of the AstraZeneca ChAdOx1 nCoV-19 vaccine in preventing symptomatic COVID-19 infection at least 14 days after the second dose and 100% efficacy in preventing hospitalization and/or death from SARS-CoV-2 (13). Further exploratory analyses have shown that vaccine efficacy between day 22 and day 90 following a single standard dose was 76.0%; effectiveness ≥14 days after the second dose among those who received a second dose of vaccine ≥12 weeks after the first dose was 81.3%. In an RCT of 44,325 healthy adults, the Janssen vaccine was 66.3% effective at preventing laboratory-confirmed symptomatic COVID-19 infection 14 days post-vaccination, 93.1% effective at preventing hospitalization for severe COVID-19 and 100% effective at preventing death due to COVID-19 (17).

## VACCINE-INDUCED IMMUNE THROMBOTIC THROMBOCYTOPENIA (VITT)

Recent rare reports of cerebral and systemic venous thromboembolic events (VTEs), associated with thrombocytopenia and hemorrhage with both the AstraZeneca and Janssen vaccines have raised concerns regarding the risk-benefit ratio of these vaccines, particularly among younger individuals who are at very low risk of dying from COVID-19 (18–20). Termed vaccine-induced immune thrombotic thrombocytopenia (VITT), the VTE that develop with VITT have been often associated with life-threatening cerebral VTE (21,22). While the rate of VITT with the AstraZeneca vaccine was initially suggested to be about 1 in 250, persons (18), more recent data have suggested that the rate may be much higher (1 in 60,000 persons, 0.0017%) (20). The risk is expected to be similar for people with IBD as the mechanism of VTE development in VITT differs from traditional VTE (22).

In light of the risk of VITT, NACI has issued a statement that mRNA vaccines are preferred over non-mRNA vaccines. These facts have also prompted several provincial health authorities to suspend administration of the AstraZeneca vaccine as the first dose until more data are available and to consider administering an mRNA vaccine for the second dose among those who received the AstraZeneca vaccine for the first dose (20,23). These policy changes have also come in the face of increasing supply of mRNA vaccines, diminishing supply of AstraZeneca vaccine and decreasing rates of COVID-19 in society.

Importantly, the background risk of VTE in the Canadian population is roughly 0.1% (24), and the risk of VTE among individuals hospitalized with COVID-19 is as high as 15% (25). Considering the adverse impacts of COVID-19, the benefits of these vaccines in preventing symptomatic and severe COVID-19 may still outweigh the potential risks for individuals who have risk factors for acquiring COVID-19 or for having an adverse outcome with COVID-19, such as those who are elderly, have multi-morbidity, or live in a region with high SARS-CoV-2 transmission rates (particularly of novel variant strains), and especially in regions where mRNA vaccines are in short supply (18).

## VACCINE PRODUCTION AND DISTRIBUTION IN CANADA

For many years, Canada was a leader in vaccine production (26). While plants in Toronto and Montreal have continued producing vaccines, they are not equipped to manufacture the COVID-19 vaccines, resulting in the Canadian government seeking vaccines from the U.S., Europe and India. (*Canadian Health Policy*, December 2020. ISSN 2562-492). However, this led to a lag in Canadian vaccine procurement and vaccination efforts when Moderna and Pfizer reduced vaccine production to build capacity for larger-scale production. Further, transport and storage logistics of existing vaccines coming from Europe and the US to a central repository in Canada (and later to regional distribution centres and provincial or territory points of care) has been challenging for some of the COVID-19 vaccines, particularly those requiring storage at −20°C to −80°C.

The Canadian government plans to increase the domestic production capability of COVID-19 vaccines (27). First, a new facility will be created for the National Research Council in Montreal, where ten million Novavax vaccines will be made, pending Health Canada approval (as of writing, the Novavax SARS-CoV-2 vaccine [NVX-CoV2373] is completing Phase 3 trials with healthy adults in the United Kingdom and is still pending approval by Health Canada [application made on January 29, 2021]; Novavax is a recombinant nanoparticle vaccine utilizing the spike glycoprotein of the SARS-CoV-2 virus). Additionally, companies that have demonstrated past success in manufacturing capabilities have received massive government investment. For example, VIDO-interact in Saskatoon has been allocated upwards of $45 million to build a manufacturing plant, and Medicago has been allocated upwards of $170 million to expand its manufacturing capacity, in addition to a specific order of 76 million doses of its vaccine, pending Health Canada approval.

## SARS-COV-2 VACCINE RECOMMENDATIONS FOR PEOPLE WITH IBD

People with chronic immune-mediated diseases such as IBD were excluded from the trials that evaluated the currently approved SARS-CoV-2 vaccines; therefore, concerns have been raised by healthcare providers and the IBD community regarding the safety and efficacy of SARS-CoV-2 vaccines for people with IBD, as well as the influence of drug therapy on vaccine immunogenicity and safety. Importantly, as none of the approved vaccines are live attenuated vaccines, there is no reason to suspect that individuals with IBD receiving immunosuppressive therapy would be at increased risk of virus reactivation. Moreover, multiple observational studies have now been published on outcomes of SARS-CoV-2 vaccination in people with IBD with no cause for concern indicated by any.

In a study of 246 people with IBD who received a SARS-CoV-2 vaccine, the overall rate of adverse events was similar to the general population, while biologic therapy was associated with fewer adverse events, possibly due to blunting of an aggressive immune response (28). A study of 1500 individuals with IBD in the UK (CLARITY-IBD) reported that infliximab treatment was associated with a less robust immune response to the first dose of the Pfizer or AstraZeneca vaccine as compared to vedolizumab. Moreover, individuals receiving concomitant azathioprine or methotrexate had reduced seroconversion with both infliximab and vedolizumab (29). Similarly, an earlier UK study of 7000 people with IBD reported that serological responses to SARS-CoV-2 infection were attenuated in individuals receiving infliximab relative to those receiving vedolizumab and further blunted by concomitant azathioprine or methotrexate (30). Overall, these findings are consistent with observations of reduced immunogenicity to other vaccines in people with IBD receiving anti-TNF therapy (alone or in combination) and traditional immunosuppressive therapies, such as azathioprine, 6 mercaptopurine, methotrexate and higher doses of corticosteroids (prednisone ≥20 mg per day), including those for pneumococcus (31–34), influenza viruses (35–39), hepatitis B virus (40), hepatitis A virus (41) and herpes zoster virus (42), Immune responses to conventional vaccines do not appear to be impacted by vedolizumab (influenza and hepatitis B) (43) or ustekinumab (influenza, pneumococcal, tetanus) (44,45).

Importantly, in the CLARITY-IBD study, seroconversion was robust following the second dose of vaccine and in individuals who received a dose of vaccine following recovery from COVID-19 infection. Furthermore, a national US cohort study of nearly 15,000 predominantly white males with IBD receiving a wide spectrum of medications in the Veterans Health Administration System reported that the risk of infection with SARS-CoV-2 was 1.34% in those who were unvaccinated and 0.11% among those who were at least seven days from their second dose of mRNA vaccine (80.4% vaccine effectiveness) (46).

Canadian (47), European (48) and international (49) organizations recommend that people with IBD be vaccinated against SARS-CoV-2 at the earliest opportunity, irrespective of vaccine type, disease status or treatment and without interruption of scheduled therapy. Crohn’s and Colitis Canada further recommends that persons with IBD receive a scheduled second dose of vaccine three to four weeks following the first dose to boost immunity, rather than extending the interval for the second dose by up to 16 weeks as for the general population. As individuals with IBD receiving immunosuppressive medications may be at higher risk of contracting severe COVID-19 than the general public (50,51), vaccination is particularly relevant in this group. NACI now “preferentially recommends that a complete two-dose vaccine series with an mRNA COVID-19 vaccine (Pfizer-BioNTech, Moderna) should be offered to individuals in the authorized age group, including those who are immunosuppressed, have an autoimmune condition, are pregnant or are breastfeeding.” Furthermore, it recommends that “with the increase of COVID-19 vaccine supply in Canada, second doses should be offered as soon as possible, with priority given to those at highest risk of severe illness and death from COVID-19 disease” (NACI updated COVID-19 vaccine statement, May 28, 2021: Summary—Canada.ca).

Regardless of vaccination status, individuals with IBD should remain vigilant with practicing recommended public health measures to prevent SARS-CoV-2 infection and transmission. Given the impaired serological responses to SARS-CoV-2 among people on immunosuppressive treatment, serological testing and virus surveillance should be considered to detect suboptimal vaccine responses, infection persistence and viral evolution to inform public health policy.

Vaccine recommendations for special populations with IBD (children and adolescents, pregnant people and seniors) are provided in articles dedicated to those populations (Benchimol, this volume; Bernstein, this volume).

## CONCLUSION

The collective efforts of scientists, physicians, politicians and industry have led to the fastest ever vaccine development program against any infectious disease known to mankind. Moreover, efficacy and safety of the approved vaccines have been extremely high. While SARS-CoV-2 variants are on the rise across the globe, we are closer to achieving herd immunity with the development and mass rollout of these efficacious vaccines. Despite people with IBD being excluded from the pivotal COVID-19 vaccine trials, emerging evidence and past evidence from other vaccination programs suggest that these vaccines should be similarly safe and effective in those with IBD. As such, the overwhelming recommendation by all societies is that individuals with IBD should receive any of the available vaccines at the earliest opportunity, without delay or interruption of their IBD treatment. Future research is needed to determine if vaccine efficacy wanes among those receiving anti-TNF therapy or conventional immunosuppressive agents and if a timely booster dose is warranted to increase protection. It is important that people with IBD are not excluded from existing vaccination programs and that they do their part to procure timely vaccination to contribute to the ultimate goal of achieving herd immunity.
